# Intestinal Epithelial-like Cells Stimulated by a Functional Food Ingredient Promote Lysyl Oxidase (LOX) Expression in Osteoblast Precursor Cells via BMP-1 Secretion

**DOI:** 10.3390/ijms27073156

**Published:** 2026-03-31

**Authors:** Naoki Fujimoto, Shotaro Suzuki, Tomohiro Yano, Shinji Sakata, Yuka Ito, Tatsuya Ishida

**Affiliations:** 1 Graduate School of Health and Sports Sciences, Toyo University, Tokyo 115-8650, Japan; s4h202400028@toyo.jp (N.F.);; 2 Asahi Quality & Innovations, Ltd., Moriya-Shi 302-0106, Ibaraki, Japan

**Keywords:** bone quality, gut–bone crosstalk, intestinal epithelial-like cells, osteoblast precursor cells, lysyl oxidase, BMP-1

## Abstract

Osteoporosis is characterized by reductions in bone mineral density (BMD) and bone quality. While gut-derived signaling has been increasingly studied in relation to BMD, its contribution to molecular factors associated with bone quality remains less defined. Here, we investigated whether a heat-inactivated, freeze-dried, non-viable preparation of *Levilactobacillus brevis* AS-1 modulates intestinal epithelial-like cells and thereby promotes lysyl oxidase (LOX), a key enzyme involved in collagen cross-linking. Caco-2 cells were treated using 1 mM sodium butyrate and subsequently stimulated with 100 μg/mL *L. brevis* AS-1. Supernatants were collected and applied to MG63 cells. Cytokine mRNA expression in Caco-2 cells and LOX responses in MG63 cells were analyzed by qRT-PCR, and bone morphogenetic protein (BMP-1) and transforming growth factor-β (TGF-β)1 protein levels in the supernatant were measured by ELISA. *L. brevis* AS-1 stimulation up-regulated BMP-1 and TGF-β1 mRNA expression in SB-treated Caco-2 cells and increased BMP-1 protein secretion into the supernatant. LOX mRNA expression and total LOX activity were increased in MG63 cells treated with the conditioned supernatant, and inhibition of BMP-1/procollagen C-proteinase activity (UK383367) attenuated LOX mRNA induction. Collectively, these results suggest that *L. brevis* AS-1 stimulates intestinal epithelial-like cells to secrete BMP-1, which in turn promotes LOX mRNA expression in osteoblast precursor cells. This in vitro mechanism supports the concept of gut–bone crosstalk regulating molecular factors associated with bone quality.

## 1. Introduction

Population aging is accelerating worldwide, increasing the burden of age-related disorders. Osteoporosis is a major contributor to fractures and loss of independence, highlighting the need for preventive strategies. Bone strength reflects both bone mineral density (BMD) and bone quality; notably, bone quality is strongly influenced by extracellular matrix maturation, including collagen cross-linking initiated by lysyl oxidase (LOX) [1,2]. LOX initiates the cross-linking process by oxidatively deaminating specific lysine and hydroxylysine residues, generating reactive aldehydes that subsequently form intra- and intermolecular cross-links, which provide a three-dimensional scaffold for matrix organization and mineral deposition [3]. LOX activity is regulated by extracellular processing enzymes such as BMP-1 [4], and LOX-dependent collagen organization is an essential determinant of osteogenesis and bone quality [5].

The gut–bone axis has been proposed to involve multiple interconnected mechanisms, including intestinal calcium absorption, microbial metabolites (e.g., short-chain fatty acids), epithelial barrier integrity, and immune-mediated pathways that regulate osteoclast–osteoblast balance [6,7]. Immune-mediated pathways, including the RANK/RANKL/OPG axis and inflammatory cytokines (e.g., TNF-α and IL-6), regulate osteoclast–osteoblast coupling and can influence osteoblast differentiation and matrix remodeling [8]. Gut-derived factors such as cytokines and microbial (e.g., SCFAs) metabolites can modulate osteoblast differentiation and extracellular-matrix gene programs through inflammatory and growth-factor signaling, thereby potentially influencing collagen maturation pathways including BMP-1–LOX–mediated cross-linking. Prior probiotic studies have suggested beneficial effects on bone mass outcomes (e.g., BMD) through modulation of inflammatory or endocrine mediators [9,10]; however, how intestinal epithelial/immune signals mechanistically influence collagen maturation pathways relevant to bone quality, including LOX-related processes, remains less defined. Although the clinical importance of bone quality independent of BMD has gained significant recognition since the NIH Consensus Statement in 2000/2005 [11], robust biomarkers and mechanistic determinants of bone quality remain limited [12].

We hypothesized that stimulation of differentiated intestinal epithelial-like cells with AS-1 increases the secretion of soluble mediators, particularly BMP-1, into the conditioned supernatant, and that these epithelial-derived factors promote LOX expression and activity in osteoblast precursor cells. To test this hypothesis, we used a two-step in vitro epithelial-to-osteoblast communication assay in which supernatant collected from Caco-2 cells was transferred to MG63 cells, followed by the evaluation of BMP-1 secretion (ELISA) and LOX responses (qRT-PCR and activity assay).

## 2. Results

### 2.1. Differentiation of Caco-2 Cells

Caco-2 cells can undergo maturation-associated changes accompanied by alterations in brush-border enzyme expression and activity. Sodium butyrate (SB) induces differentiation and apoptosis [13] and has been shown to affect cytokine secretion, nuclear factor kappa B (NF-κB) signaling, and alkaline phosphatase (ALP) activity in colon cancer cells [14].

**Figure 1:**
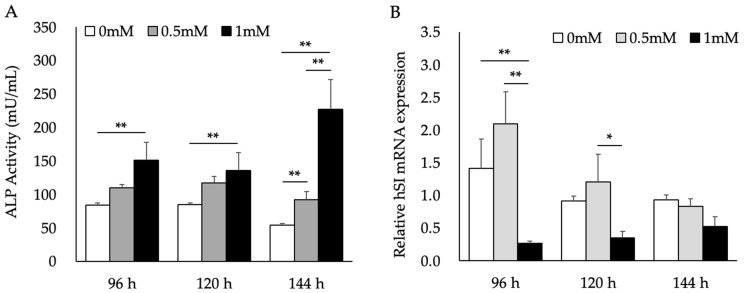
Effects of 0, 0.5, and 1 mM sodium butyrate (SB) on Caco-2 cells treated for 96, 120, and 144 h. (**A**) ALP activity was analyzed using the Amplite™ Alkaline Phosphatase Assay Kit. (**B**) hSI mRNA levels were analyzed by qRT-PCR and normalized to RPL32. Data are presented as the mean ± standard deviation (SD) (*n* = 3 independent biological replicates). * *p* < 0.05, ** *p* < 0.01; One-way ANOVA followed by Tukey’s multiple comparisons test.

To examine the effects of *L. brevis* AS-1 under our SB-based conditioning protocol, we pretreated Caco-2 cells with SB prior to AS-1 exposure. ALP activity was significantly increased by 1 mM SB (Figure 1A) and was used here as the primary functional indicator of SB-induced maturation-associated changes. In contrast, at 96 h human sucrase–isomaltase (hSI) mRNA expression was significantly decreased in the 1 mM SB group compared with the 0 mM control, whereas the 0.5 mM SB group showed a higher mean value that did not reach statistical significance (Figure 1B). Taken together, these results indicate that SB induced maturation-associated changes in Caco-2 cells under our experimental conditions, as evidenced primarily by increased ALP activity.

### 2.2. Increases in LOX mRNA Expression and Activity in MG63 Cells Treated with the Supernatant (SP) of SB-Treated Caco-2 Cells

**Figure 2:**
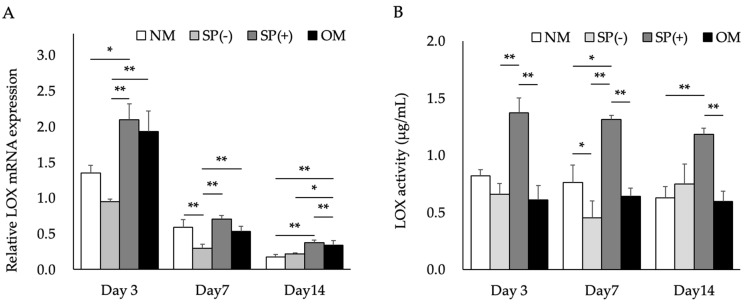
Effects of normal medium (NM), supernatant from SB-treated Caco-2 cell showing maturation-associated changes prepared with *L. brevis* AS-1 (SP(+)) or without AS-1 (SP(−)), and osteoblast inducer medium (OM) on LOX mRNA expression (**A**) and activity (**B**) in MG63 cells cultured for 3, 7, and 14 days. SP(+) was defined as the supernatant collected from Caco-2 cells treated with 1 mM SB and stimulated with 100 µg/mL *L. brevis* AS-1; SP(−) was prepared identically but without AS-1. All supernatants were supplemented with 2% FBS before application to MG63 cells, and the medium was replaced every 3 days with the corresponding medium. LOX mRNA expression was analyzed by qRT-PCR and normalized to RPL32. LOX activity was analyzed using the Fluorometric Lysyl Oxidase Assay Kit. Data are presented as the mean ± SD (*n* = 3 independent biological replicates). * *p* < 0.05, ** *p* < 0.01; One-way ANOVA followed by Tukey’s multiple comparisons test.

We investigated LOX mRNA expression and activity in MG63 cells treated with the SP of SB-treated Caco-2 cells with (SP(+)) and without *L. brevis* AS-1 (SP(−)). LOX mRNA expression and activity were higher in MG63 cells cultured in osteoblast inducer medium (OM) than in control cells. LOX mRNA expression was significantly up-regulated in MG63 cells treated with SP(+), whereas its mRNA expression and activity remained unchanged in those treated with SP(−). Although absolute LOX mRNA expression decreased over time, LOX mRNA expression remained higher in SP(+)-treated cells than in SP(−)-treated cells across the time points examined (Figure 2A). LOX activity was significantly higher in SP(+)-treated cells than in control cells (Figure 2B). Furthermore, LOX mRNA expression and activity were higher in SP(+)-treated cells than in OM-treated cells.

### 2.3. Effects of Cytokines Induced by L. brevis AS-1 in SB-Treated Caco-2 Cells

**Figure 3:**
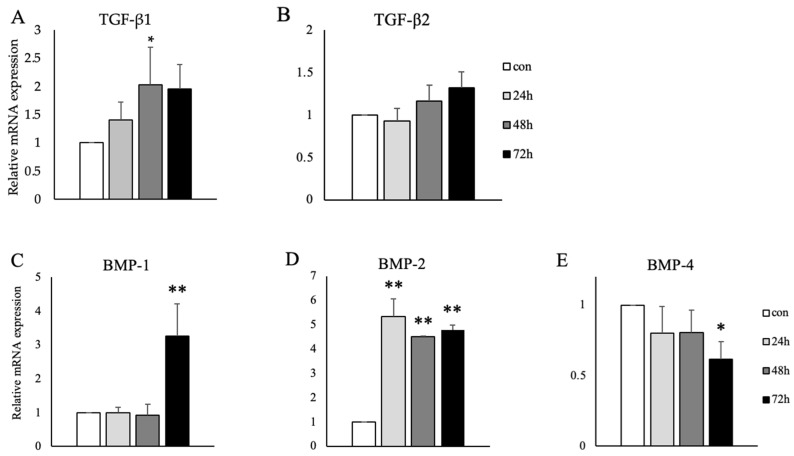
Effects of *L. brevis* AS-1 on cytokine/growth factor-related mRNA expression in Caco-2 cells treated for 24, 48, and 72 h. mRNA expression was analyzed by qRT-PCR and normalized to RPL32. (**A**) TGF-β1, (**B**) TGF-β2, (**C**) BMP-1, (**D**) BMP-2, and (**E**) BMP-4 mRNA expression. Data are presented as the mean ± SD (*n* = 3 independent biological replicates). * *p* < 0.05, ** *p* < 0.01; One-way ANOVA followed by Dunnett’s multiple comparisons test (vs. control).

To examine proteins that induce LOX mRNA expression, we measured transforming growth factor-β (TGF-β)1/2 and bone morphogenetic protein (BMP)-1/2/4 mRNA expression levels in SB-treated Caco-2 cells. Figure 3 shows that *L. brevis* AS-1 significantly increased TGF-β1, BMP-1 and BMP-2 mRNA expression levels in SB-treated Caco-2 cells (Figure 3A,C,D). No significant change was observed in TGF-β2 mRNA expression levels (Figure 3B), while BMP-4 mRNA expression levels significantly decreased (Figure 3E).

### 2.4. Effects of Cytokines Secreted from MG63 Cells on Signal Pathways Regulating LOX

**Figure 4:**
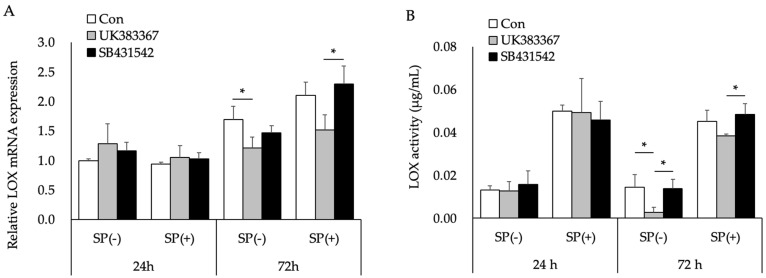
Effects of UK383367 and SB431542 on LOX mRNA expression (**A**) and activity (**B**) in MG63 cells cultured in the supernatant of SB-treated Caco-2 cells treated with (SP(+)) or without *L. brevis* AS-1 (SP(−)). LOX mRNA expression was analyzed by qRT-PCR and normalized to RPL32, and LOX activity was measured using the Fluorometric Lysyl Oxidase Assay Kit. Data are presented as the mean ± SD (*n* = 3 independent biological replicates). * *p* < 0.05; One-way ANOVA followed by Tukey’s multiple comparisons test (within each time × SP condition).

Figure 4 shows the effects of TGF-β type I receptor kinase (ALK5) inhibitor (SB431542) and BMP-1/procollagen C-proteinase inhibitor (UK383367) on LOX mRNA expression and activity. Neither inhibitor affected LOX mRNA expression or activity after 24 h (Figure 4A,B), while UK383367 significantly decreased LOX mRNA expression after 72 h (Figure 4A). SB431542 did not affect LOX mRNA expression or activity at any time point examined (Figure 4A,B). The raw fluorescence intensity values before blank subtraction, from which the LOX activity data shown in Figure 4B were derived after standard-curve conversion, are provided in Supplementary Figure S4.

### 2.5. Estimation of Signal Pathways Related to BMP-1 Expression in SB-Treated Caco-2 Cells Treated with L. brevis AS-1

**Figure 5:**
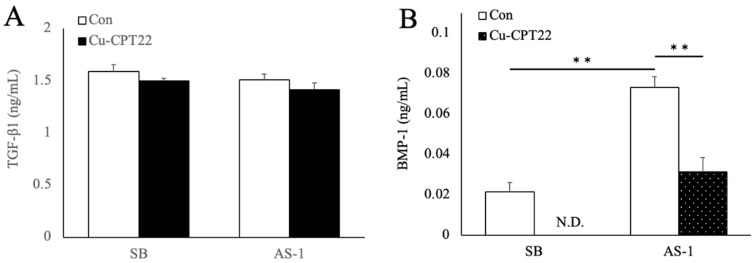
Responses of Caco-2 cells against *L. brevis* AS-1 treated with Cu-CPT22. TGF-β1 (**A**) and BMP-1 (**B**) concentrations were measured by ELISA in SB-treated Caco-2 cells treated with SB alone or with the combination of *L. brevis* AS-1 and SB, each with or without Cu-CPT22. Data are presented as the mean ± SD (*n* = 3 independent biological replicates). N.D., not detected. Values below the lower limit of quantification (LLOQ; 0.16 ng/mL, the lowest standard concentration) were treated as <LLOQ and plotted as 0 for visualization. ** *p* < 0.01; One-way ANOVA followed by Tukey’s multiple comparisons test.

We examined the effects of a toll-like receptor (TLR) inhibitor (Cu-CPT22) on BMP-1 and TGF-β1 secretion in SB-treated Caco-2 cells treated with *L. brevis* AS-1. Cu-CPT22 did not inhibit the *L. brevis* AS-1-induced secretion of TGF-β1 in SB-treated Caco-2 cells (Figure 5A). *L. brevis* AS-1 significantly promoted and Cu-CPT22 significantly inhibited the secretion of BMP-1 in these cells (Figure 5B). In parallel, MG63 LOX mRNA expression and activity were increased by SP(+) but not by SP(−) (Figure 2), and LOX mRNA expression was reduced by the BMP-1/procollagen C-proteinase inhibitor UK383367 (Figure 4).

## 3. Discussion

The relationship between bone and the gut microbiota has been attracting increasing attention in recent years [15]. While the mechanisms by which the gut microbiota influence BMD are still being investigated, the gut–bone axis is thought to involve multiple routes, including intestinal epithelial barrier signaling, microbial metabolites, and (in vivo) immune-mediated pathways [16,17]. Collins et al. suggested the modulation of gut-derived host signaling may represent a therapeutic avenue for osteoporosis, as regulation of osteoclasts and osteoblasts by gut-related factors has been associated with increased bone mass [9]. Nilsson et al. reported that *L. reuteri* may reduce bone loss in older women with low BMD [18]. Based on these findings, the prevention of osteoporosis via gut-derived factors using functional food ingredients is promising. Increases in both BMD and bone quality are important for the prevention of osteoporosis [1]. In the present study, we examined improvements in bone quality using an in vitro epithelial-to-osteoblast communication model in which intestinal epithelial-like cells were stimulated with *L. brevis* AS-1 and the conditioned supernatant was applied to osteoblast precursor cells to probe gut–bone crosstalk at the level of epithelial-derived signaling.

Figure 1 shows that SB induced maturation-associated changes in Caco-2 cells, as reflected by alterations in brush-border enzyme-related readouts. ALP activity and sucrase–isomaltase (hSI) expression are commonly used indicators of Caco-2 differentiation, because Caco-2 differentiation is characterized by increased brush-border enzyme activities such as ALP and SI [19]. Sodium butyrate has been widely used to promote differentiation-associated maturation of Caco-2 cells and is a known HDAC inhibitor, supporting an epigenetic basis for butyrate-induced changes in intestinal functional programs [20,21]. Previous studies further indicate that cellular response to butyrate depends on basal differentiation status and are associated with histone hyperacetylation together with induction of differentiation-related endpoints including ALP activity [22]. In the present study, SB robustly increased ALP activity, indicating an enhancement of brush-border enzyme activity/maturation in this model; however, hSI mRNA decreased under our experimental conditions, consistent with a context-dependent transcript response. Therefore, we used ALP activity as the primary functional indicator of SB-induced maturation-associated changes, and interpreted hSI as a secondary transcript readout. Importantly, the final SB concentration delivered to MG63 cells was matched across all supernatant groups to minimize potential direct effects of residual SB on the downstream LOX readouts.

SB is a histone deacetylase (HDAC) inhibitor and has been reported to engage cell-cycle regulatory programs (including p53–p21–linked pathways) associated with G1 arrest and differentiation/maturation-associated phenotypic changes in Caco-2 cells [23]. Consistent with this concept, SB has been reported to promote Caco-2 differentiation in a cell type–specific manner, accompanied by reduced expression of cell-cycle–related genes (e.g., cyclin B1) [24]. Thus, the SB-induced increase in ALP activity together with the condition-dependent change in hSI mRNA observed here is interpreted as being consistent with SB-driven maturation-associated remodeling of enterocyte-like functions.

Figure 2 shows the results obtained on LOX mRNA expression and activity in MG63 cells treated with the SP of SB-treated Caco-2 cells. *L. brevis* AS-1 appeared to increase the mRNA expression and activity of LOX, consistent with the role of LOX in collagen cross-links for bone quality [1]. Turecek et al. reported that removal of the extracellular matrix decreased bone formation markers, indicating that extracellular components, such as collagen and LOX, are necessary for osteoblast differentiation [2]. Ida et al. demonstrated that even when LOX mRNA expression and collagen production increased, osteoblast differentiation did not proceed unless LOX formed cross-links [25]. These findings support the concept that the organization of collagen fibers of the extracellular matrix by LOX is a crucial regulator of osteoblast bone formation [26]. Importantly, SP(+) and SP(−) were generated under identical SB conditions, and the final SB exposure to MG63 cells was matched across the supernatant groups. Thus, the selective LOX response to SP(+) supports an AS-1–dependent effect beyond residual SB.

The present results showed that *L. brevis* AS-1 significantly up-regulated TGF-β, BMP-1 and BMP-2 mRNA expression (Figure 3A,C,D). TGF-β, BMP-1, and BMP-2 have been shown to affect LOX mRNA expression [27,28,29]. In the fibrotic process, TGF-β1/Smad3 signaling drives the significant up-regulation of LOX mRNA and protein expression and increases its activity in fibroblasts and osteoblasts [30]. Although AS-1 increased BMP-1 and BMP-2 mRNA expression, BMP-4 mRNA showed a modest decrease (significant at 72 h). Such divergent regulation among BMP family members is plausible because inflammatory/innate immune signaling can bias BMP outputs in a gene-specific manner rather than uniformly activating all BMP ligands [31,32]. For example, oxidized LDL has been reported to induce BMP-2 and osteogenic readouts (including ALP) via TLR2/4 signaling in vascular endothelial cells, supporting the concept of preferential BMP-2 induction in innate immune–linked contexts [31,33]. We did not determine the mechanism responsible for BMP-4 downregulation in the present study; possible explanations include isoform-specific transcriptional control and/or feedback regulation within the BMP/TGF-β signaling network, which should be addressed in future work [32].

Figure 4 shows the effects of TGF-β and BMP on LOX mRNA expression and activity. SB431542 did not affect LOX expression or activity, while UK383367 significantly decreased LOX mRNA expression and slightly reduced its activity. Taken together, these results suggest that among the cytokines whose secretion from SB-treated Caco-2 cells was promoted by *L. brevis* AS-1, BMP-1 increased LOX mRNA expression and activity in MG63 cells. The inhibition of BMP-1 significantly down-regulated LOX mRNA expression, whereas its activity remained unchanged. Since LOX is secreted and functions after binding to the extracellular matrix, changes in intracellular gene expression may not be immediately reflected in total extracellular enzyme activity. Therefore, although UK383367 clearly reduced LOX mRNA expression, this effect was not directly reflected in its enzymatic activity under our assay conditions.

Figure 5A shows the effects of TLR2 signaling on the secretion of TGF-β and BMP-1. TGF-β secretion was not affected by *L. brevis* AS-1 or Cu-CPT22. Although *L. brevis* AS-1 significantly promoted BMP-1 secretion, Cu-CPT22 significantly suppressed it (Figure 5B). These results indicate that *L. brevis* AS-1 did not induce TGF-β secretion because it does not involve TLR2 in SB-treated Caco-2 cells. TLR2 recognizes lipoteichoic acid and peptidoglycan, which are major cell wall components of Gram-positive bacteria, such as lactic acid bacteria (LAB) [34]. TLR2 functions as a pattern-recognition receptor that detects multiple microbial components and initiates downstream innate immune signaling. TLR1 and TLR6 form heterodimers with TLR2. TLR1 recognizes triacyl lipopeptides [35], while TLR6 recognizes diacyl lipopeptides [36]. TLR4 recognizes lipopolysaccharide and lipoteichoic acid as cell wall components of Gram-negative bacteria [37,38]. *L. brevis* AS-1 may include lipoteichoic acid and peptidoglycans and these may be ligands of receptors in SB-treated Caco-2 cells. A limitation of this study is that AS-1 is a complex, non-viable bacterial preparation and the specific components responsible for TLR engagement were not identified. Future studies will address this by fractionation and testing of purified candidate ligands and/or TLR-specific reporter assays to determine the active components driving BMP-1 induction. When considered together with the MG63 data showing a selective LOX response to SP(+) (Figure 2) and the suppression of LOX mRNA expression by the BMP-1/procollagen C-proteinase inhibitor UK383367 (Figure 4), the ELISA results (Figure 5B) support the interpretation that TLR1/2-dependent BMP-1 secretion from SB-treated Caco-2 cells is a mechanistic contributor to the downstream LOX upregulation in MG63 cells.

In addition, we provide the effects of pharmacological inhibition of TLR2 (C29) and TLR4 (TAK-242) on AS-1-responsive cytokine transcripts in SB-treated Caco-2 cells (Supplementary Figure S3). Under these conditions, both inhibitors showed a numerical tendency to attenuate AS-1–associated BMP-1 and TGF-β1 mRNA levels, although the differences versus AS-1 alone did not reach statistical significance in this dataset. The TLR1/2 inhibitor Cu-CPT22 markedly suppressed AS-1–induced BMP-1 secretion (Figure 5B), supporting involvement of TLR1/2-linked epithelial signaling in regulating BMP-1 output. Therefore, further studies are needed to investigate the effects of inhibiting TLR1/2 and TLR2/6 on BMP-1 secretion. In the present study, *L. brevis* AS-1 appeared to promote BMP-1 secretion via TLR1/2 in SB-treated Caco-2 cells, and BMP-1 increased LOX mRNA expression and activity in MG63 cells.

Many probiotic/LAB studies on bone health have primarily evaluated bone mass–related outcomes (e.g., BMD, microarchitecture, and bone turnover markers) and have often discussed benefits in the context of systemic immune modulation and/or endocrine and absorption-related pathways [39,40,41,42]. In contrast, our epithelial-to-osteoblast communication model suggests a complementary route in which stimulation of SB-treated Caco-2 cells with a non-viable bacterial preparation increases BMP-1 secretion and engages a matrix-maturation pathway relevant to bone quality via LOX-related processes. This distinction is important because bone strength reflects both bone mass and matrix organization, and matrix-related improvements may occur with modest effects on BMD [24].

The gut–bone axis is frequently discussed in terms of cytokine networks that regulate osteoclastogenesis and osteoblast activity, including the RANKL/OPG axis and inflammatory mediators (e.g., TNF-α, IL-6, and IL-17) counterbalanced by anti-inflammatory pathways (e.g., IL-10 and TGF-β) [17,43,44]. Our findings do not exclude these established frameworks; rather, they indicate that epithelial-derived BMP-1 may represent an additional mediator linking gut-associated signaling to extracellular matrix maturation programs relevant to bone quality. Consistent with this concept, BMP-1 is a procollagen C-proteinase involved in extracellular matrix processing, providing a plausible connection to collagen cross-linking pathways [45,46].

Reported probiotic effects on bone are heterogeneous and sometimes contradictory, which is plausible given context dependence (strain specificity, viability/formulation, dose and duration, host factors, and endpoint selection) [39,40,41,42]. Interventions that primarily modulate systemic inflammation may preferentially affect osteoclast-related outputs, whereas interventions that alter epithelial-derived mediators may influence matrix-related programs [16,17,43,44]. In line with context-dependent signaling, bone-regulating cytokines can be regulated in a gene-specific manner rather than uniformly [17,43]; in our dataset, AS-1 increased BMP-1 (and BMP-2 transcripts) but did not uniformly upregulate all BMP family members, and TGF-β secretion was not increased under our conditions. Such selective outputs are compatible with the idea that innate/epithelial signaling can bias downstream mediator profiles [32]. Future studies should test how epithelial BMP-1 output integrates with canonical osteoclast-regulating mediators and inflammatory cytokines in more physiologic systems [17,43,44].

The gut microbiota and LAB are known to induce various immune-related factors through TLR2; however, limited information is currently available on LAB strains that enhance BMP-1 expression via TLR2 signaling. BMP-1 has also been shown to increase LOX protein levels [26]. Therefore, the up-regulation of BMP-1 by *L. brevis* AS-1 may represent a novel mechanism by which intestinal epithelial–bone crosstalk contributes to improvements in bone quality.

We focused on LOX as a representative cross-linking enzyme; however, other LOX family members (LOXL1–LOXL4) and collagen degradation pathways (MMP/TIMP) may also contribute to matrix maturation and should be assessed in future studies [24]. Because this work is based on an in vitro epithelial-to-osteoblast communication assay using SB-treated, carcinoma-derived Caco-2 and MG63 cell lines, the findings should be interpreted as mechanistic evidence; MG63 cells may differ from primary osteoblasts in collagen/ECM programs and growth-factor responsiveness, which could influence LOX-related readouts. Although phenol red (a weak estrogen mimic) was present in the MEM used across all conditions, SP(−) and SP(+) were generated and applied under identical base-medium and serum conditions; thus, it is unlikely to account for between-group differences, although confirmation under phenol red–free conditions would strengthen translatability. We did not directly measure the absolute pH or nutrient composition of conditioned supernatants; future studies should quantify these parameters and/or include pH-matched controls to further exclude non-specific stress effects. Key findings should be validated in non-cancerous human osteoblast models (primary human osteoblasts or human MSC-derived osteoblasts) and more physiologic systems. The working concentration of AS-1 (100 µg/mL) was selected for reproducibility and physiological/clinical plausibility, but dose–response relationships (including BMP-1 secretion) and potential batch-to-batch variability should be evaluated using multiple independent production lots. Because AS-1 is a non-viable, sonicated postbiotic preparation, mass-based dosing cannot be directly converted to CFU equivalents.

Collectively, the present results indicate that the functional food ingredient *L. brevis* AS-1 up-regulated LOX mRNA expression in osteoblast cells via intestinal epithelial cells in our in vitro model, and a possible mechanism was suggested. The use of gut-derived epithelial signaling may contribute to the development of new osteoporosis prevention methods that target bone quality.

## 4. Materials and Methods

### 4.1. Reagents and Materials

All culture reagents and SB were purchased from Nacalai Tesque (Kyoto, Japan), unless otherwise indicated. Fetal bovine serum (FBS) was purchased from Bio West (Nuaillé, France). The Amplite^TM^ Colorimetric Alkaline Phosphatase Assay Kit *Yellow Color* and Amplite^TM^ Fluorimetric Lysyl Oxidase Assay Kit *Red Fluorescence* were purchased from AAT Bioquest (CA, USA). The TGF-β1 ELISA Kit, Quantikine, 2nd Generation was purchased from R&D SYSTEMS (MN, USA). The Human BMP-1/PCP ELISA Kit was purchased from Novus Biologicals (CO, USA).

*L. brevis* AS-1 was provided by Asahi Quality & Innovations, Ltd. (Ibaraki, Japan). AS-1 was used as a non-viable, freeze-dried preparation (heat-inactivated at 121 °C for 15 min prior to freeze-drying). For stimulation assays, the freeze-dried preparation was reconstituted and then sonicated to disrupt cells and homogenize bacterial components. All experiments were performed using a single production lot of AS-1. OM was purchased from Takara Bio Inc. (Shiga, Japan). The TGF-β type I receptor kinase (ALK5) inhibitor (SB431542) and TLR1/2 inhibitor (Cu-CPT22) were purchased from Selleck Biotech (Kanagawa, Japan). TLR2 inhibitor (C29) and TLR4 inhibitor (TAK-242) were purchased from Nacalai Tesque (Kyoto, Japan). Working concentrations of inhibitors were determined based on WST-8–based cell viability assays (Cell Counting Kit-8; Supplementary Figure S2).

SB was dissolved in phosphate-buffered saline (PBS) to 10 mM, suspended by vortexing, and sterilized using a 0.22-µm sterile filter (Millex^®^-GS, 0.22 µm, Merck KGaA, Hesse, Germany).

The *L. brevis* AS-1 sample was dissolved in 10 mM SB solution to 1 mg/mL, vortexed (1 min), sonicated (5 min) (UT-206, SHARP, Osaka, Japan), and vortexed again (1 min). The combination of *L. brevis* AS-1 and SB medium consisted of 2.8 mL of modified Eagle’s medium (MEM), 350 μL of FBS, and 10 mM SB supplemented with 350 μL of *L. brevis* AS-1. The SB medium contained 2.8 mL of MEM, 350 μL of FBS, and 10 mM SB. The working concentrations of SB, UK383367, SB431542, and Cu-CPT22 used in this study were determined based on WST-8–based cell viability assays, as shown in Supplementary Figure S2.

### 4.2. Cell Culture

The Caco-2 human colon cancer epithelial cell line and the MG63 human osteosarcoma cell line were purchased from Riken BRC (Ibaraki, Japan), cultured in MEM, and supplemented with 10% FBS, 0.5% Penicillin–Streptomycin, D-(+)-glucose, L-Glutamine, and Phenol Red at 37 °C in a humidified atmosphere with 5% CO_2_. Cells were plated on culture plates and cultured for 48 h (Caco-2 cells) and 24 h (MG63 cells) to permit adherence. After Caco-2 cells had grown to 80% confluence, the original cell culture medium was discarded, and 1 mM SB or PBS (control) was added. Caco-2 and MG63 cells were used between passages P2 and P10 and were passage-matched as closely as possible across experimental conditions. Cells were routinely screened for mycoplasma contamination using a PCR-based detection method.

### 4.3. Differentiation of Caco-2 Cells

#### 4.3.1. ALP Activity Assay

Caco-2 cells were cultured at a density of 1.5 × 10^5^ cells/mL in a 60 mm dish for 48 h, and were then treated with SB for 96, 120, and 144 h. After the treatment, the SP was collected. The ALP activity was measured as a commonly used brush-border enzyme activity marker of Caco-2 maturation-associated changes using the Amplite™ Colorimetric Alkaline Phosphatase assay kit (AAT Bioquest, CA, USA). The assay was performed according to the manufacturer’s protocol. Absorbance was measured at 410 nm using a multi-well plate reader (SUNRISE Rainbow RC-R, Tecan Japan, Kanagawa, Japan).

#### 4.3.2. Assessment of HSI mRNA Expression in Caco-2 Cells by Quantitative Real-Time PCR (qRT-PCR)

Caco-2 cells were cultured at a density of 1.5 × 10^5^ cells/mL in a 60 mm dish for 48 h, and were then treated with SB for 96, 120, and 144 h. Total RNA was isolated from Caco-2 cells using a Tissue Total RNA Extraction Mini Kit (Favorgen Biotech Corp., Ping-Tung, Taiwan). Total RNA (100 ng for each sample) was used for cDNA synthesis using the ReverTra Ace qPCR RT Kit (Toyobo, Osaka, Japan). The cDNA template was analyzed by real-time PCR using the ABI Prism 7000 Sequence Detection System (Applied Biosystems Japan Ltd., Tokyo, Japan) and THUNDER-BIRD™ SYBR qPCR Mix (Toyobo, Osaka, Japan) according to the following program: at 95 °C for 10 s, followed by 40 cycles at 95 °C for 15 s and 60 °C for 1 min. Primer sets are shown in Table 1. Gene expression data were normalized to the expression of the reference gene ribosomal protein L32 (RPL32).

### 4.4. Assessment of mRNA Expression of LOX in MG63 Cells by qRT-PCR

After 48 h of the SB treatment, the medium was removed and replaced with fresh medium containing AS-1 and SB or SB alone. Cells were then cultured for 96 h. SP from Caco-2 cells treated with or without *L. brevis* AS-1 [SP(+) and SP(−)] was collected and centrifuged (800× *g*, 20 °C, 3 min). FBS was added to each SP to achieve a final concentration of 2% prior to application to MG63 cells, and both SP(+) and SP(−) were applied to MG63 cells under the same 2% FBS condition. OM was adjusted to contain ascorbic acid, hydrocortisone, and β-glycerophosphate at 1% (*v*/*v*), 0.2% (*v*/*v*), and 2% (*v*/*v*), respectively. MG63 cells were cultured in OM or the collected SP for 3, 7, or 14 days.

To investigate the effects of cytokines on LOX mRNA expression and activity, 1 μM UK383367 (a BMP-1/procollagen C-proteinase inhibitor) or 5 μM SB431542 (a TGF-β type I receptor kinase (ALK5) inhibitor) was mixed with SP immediately before application to MG63 cells, and MG63 cells were cultured for 24 or 72 h.

Total RNA was isolated from MG63 cells using a Tissue Total RNA Extraction Mini Kit (Favorgen Biotech Corp., Ping-Tung, Taiwan). Total RNA (100 ng for each sample) was used for cDNA synthesis using the ReverTra Ace qPCR RT Kit (Toyobo, Osaka, Japan). The cDNA template was analyzed by real-time PCR using the ABI Prism 7000 Sequence Detection System (Applied Biosystems Japan Ltd., Tokyo, Japan) and THUNDER-BIRD™ SYBR qPCR Mix (Toyobo, Osaka, Japan), according to the following program: at 95 °C for 10 s, followed by 40 cycles at 95 °C for 15 s and 60 °C for 1 min. Primer sets are shown in Table 1. Gene expression data were normalized to the expression of the reference gene RPL32. A schematic overview of the experimental cell-culture workflow, including the preparation of SP from Caco-2 cells and its application to MG63 cells, is shown in Supplementary Figure S1.

### 4.5. LOX Activity Assay

The SP of MG63 cells treated with the SP of *L. brevis* AS-1- or SB-treated Caco-2 cells was collected. The Amplite^®^ Fluorimetric Lysyl Oxidase Assay kit (AAT Bioquest, 15255) was used to measure total LOX activity in SP. Increases in fluorescence in a black 96-well plate were monitored using a fluorescence microplate reader (Infinite^®^ M1000 PRO, Tecan Japan, Kanagawa, Japan) at Ex/Em = 540/590 nm.

### 4.6. Assessment of mRNA Expression of Cytokines in Caco-2 Cells by qRT-PCR

After the SB treatment for 48 h in Caco-2 cells, the culture medium was discarded, the combination of *L. brevis* AS-1 and SB or SB was added, and cells were cultured for 24, 48, and 72 h. Total RNA was isolated from Caco-2 cells using a Tissue Total RNA Extraction Mini Kit (Favorgen Biotech Corp., Ping-Tung, Taiwan). Total RNA (100 ng for each sample) for cDNA synthesis using the ReverTra Ace qPCR RT Kit (Toyobo, Osaka, Japan). The cDNA template was analyzed by real-time PCR using the ABI Prism 7000 Sequence Detection System (Applied Biosystems Japan Ltd., Tokyo, Japan) and THUNDER-BIRD™ SYBR qPCR Mix (Toyobo, Osaka, Japan), according to the following program: at 95 °C for 10 s, followed by 40 cycles at 95 °C for 15 s and 60 °C for 1 min. Primer sets are shown in Table 1. Gene expression data were normalized to the expression of the reference gene RPL32. To evaluate the involvement of TLR signaling in AS-1–induced cytokine gene expression, SB induced maturation-associated changes in Caco-2 cells were stimulated with AS-1 in the presence or absence of C29 (TLR2 inhibitor, 5 μM) and/or TAK-242 (TLR4 inhibitor, 1 μM). Cells were treated with Control, AS-1, AS-1 + C29, AS-1 + TAK-242, or AS-1 + C29 + TAK-242, and total RNA was collected 48 h after treatment. BMP-1 and TGF-β1 mRNA expression levels were analyzed by qRT-PCR and normalized to RPL32 (Supplementary Figure S3).

To verify amplification specificity, melting-curve analysis was performed after each qPCR run, and a single peak was confirmed for each primer pair. In addition, the reference gene primer set (RPL32) spans an exon–exon junction (Table 1), which reduces the likelihood of amplification from contaminating genomic DNA.

### 4.7. TGF-β1 ELISA Assay

The SP(+) and SP(−) prepared as described in Section 4.4 were collected after 96 h of culture and used for ELISA. To examine the involvement of TLR1/2 signaling in AS-1–induced cytokine secretion, Cu-CPT22 (TLR1/2 inhibitor; 5 μM) was added to the Caco-2 culture medium during the 96 h AS-1 stimulation period, and SP was collected under the same conditions. TGF-β1 levels in SP were measured using commercially available ELISA kits according to the manufacturer’s protocol (R&D SYSTEMS, MN, USA). Twenty microliters of 1 N HCl was added to 100 μL of SP and incubated at room temperature for 10 min. The reaction was then neutralized by the addition of 20 μL of 1.2 N NaOH/0.5 M HEPES (activated samples). Activated samples were diluted with 50 μL of Assay Diluent RD1-21 and then incubated at room temperature for 2 h. The plate was washed, and a detection antibody was added and incubated at room temperature for 2 h. The plates were washed again, 100 μL of the substrate solution was added, and plates were incubated at room temperature for 2 h in the dark. One hundred microliters of the stop solution was added and optical density (OD) was measured at 450 nm using a multi-well plate reader (Infinite^®^ M1000 PRO, Tecan Japan, Kanagawa, Japan). TGF-β1 concentrations were quantified using the kit-provided standard curve. The standard curve range was 31.3–2000 pg/mL, and values outside this range were considered below the lower limit of quantification (LLOQ) or above the upper limit of quantification, respectively.

### 4.8. BMP-1 ELISA Assay

The SP(+) and SP(−) prepared as described in Section 4.4 were collected after 96 h of culture and used for ELISA. To examine the involvement of TLR1/2 signaling in AS-1–induced cytokine secretion, Cu-CPT22 (TLR1/2 inhibitor; 5 μM) was added to the Caco-2 culture medium during the 96 h AS-1 stimulation period, and SP was collected under the same conditions. BMP-1 levels in the SP were measured using commercially available ELISA kits according to the manufacturer’s protocol (Novus Biologicals, CO, USA). One hundred microliters of SP was incubated at 37 °C for 90 min. Liquid from each well was removed and 100 μL of Biotinylated Detection Ab Working Solution was added. After an incubation at 37 °C for 60 min, the liquid was removed. Plates were washed using Wash Buffer and 100 μL/well of horseradish peroxidase (HRP) Conjugate Working Solution was added. After an incubation at 37 °C for 30 min, HRP Conjugate Working Solution was removed and plates were washed. Substrate Reagent at 90 µL/well was added and plates were incubated at 37 °C for 15 min in the dark. Fifty microliters of stop solution was added and OD was measured at 450 nm using a multi-well plate reader (Infinite^®^ M1000 PRO, Tecan Japan, Kanagawa, Japan). BMP-1 concentrations were quantified using the kit-provided standard curve. The standard curve range was 0.16–10 ng/mL, and values below 0.16 ng/mL were considered below the lower limit of quantification (LLOQ).

### 4.9. Statistical Analysis

Data are presented as means ± standard deviation (SD). Unless otherwise stated, all in vitro experiments were performed with *n* = 3 independent biological replicates per condition. Statistical analyses were conducted in R (version 4.4.0 (24 April 2024)). For comparisons among multiple groups, normality of model residuals was assessed using the Shapiro–Wilk test, and homogeneity of variances was evaluated using Levene’s test. When assumptions were satisfied, differences among groups were analyzed by one-way analysis of variance (ANOVA). For all-pairs comparisons among multiple groups, Tukey’s post hoc test was applied. When variances were unequal, Welch’s one-way ANOVA was used followed by the Games–Howell post hoc test. For comparisons versus a single control group (many-to-one comparisons), Dunnett’s post hoc test was performed. When a non-parametric approach was required, the Kruskal–Wallis test followed by Dunn’s test with Bonferroni correction was used. Adjusted *p*-values were used for multiple comparisons, and *p* < 0.05 was considered statistically significant. Statistical significance in figures is indicated as * *p* < 0.05 and ** *p* < 0.01. For factorial experimental designs (e.g., time × condition), analyses were performed within each predefined stratum when the primary objective was to compare treatments within that stratum.

## Figures and Tables

**Table 1:** List of PCR Primers.

Gene	Primer	Sequence	Accession (RefSeq mRNA)	Product Length (bp)	Exon-Spanning Status
*RPL32*	Forward	CATCTCCTTCTCGGCATCA	NM_000994.4	153	Exon–exon junction-spanning : Yes
	Reverse	AACCCTGTTGTCAATGCCTC			
*hSI*	Forward	CATCCTACCATGTCAAGAGCCA	NM_001041.4	196	Exon–exon junction-spanning : Yes
	Reverse	GCTTGTTAAGGTGGTCTGGTTT			
*TGF-β1*	Forward	AGGACTGCGGATCTCTGTGT	NM_000660.7	196	Intron-spanning : No
	Reverse	AGTGCCCAAGGTGCTCAATA			
*TGF-β2*	Forward	ATGCCCGTATTTATGGAGTT	NM_001135599.4	234	Intron-spanning : Yes
	Reverse	ATTGTCATTTTGGTCTTGCC			
*BMP-1*	Forward	ACGTTTCCATCGTTCGTGAG	NM_001199.4	448	Exon–exon junction-spanning : Yes
	Reverse	ACCTCCACATAGTCGTACCA			
*BMP-2*	Forward	CCAAACACAAACAGCGGAAA	NM_001200.4	159	Intron-spanning : No
	Reverse	GATGATCAGCCAGAGGAAAAGG			
*BMP-4*	Forward	TGACCACCTCAACTCAACCAA	NM_001202.6	183	Intron-spanning : No
	Reverse	CACCCACATCCCTCTACTACCA			
*LOX*	Forward	CAGAGGAGAGTGGCTGAAGG	NM_002317.7	224	Intron-spanning : Yes
	Reverse	CCAGGTTAGCTGGGGTTTACA			

Accession numbers indicate the representative RefSeq mRNA transcripts used to calculate the expected amplicon lengths. The primer sets may also match additional transcript variants; product lengths are reported based on the indicated reference transcript. Exon-spanning status was assessed in silico using UCSC In-Silico PCR (hg38). Primer pairs that produced amplicons in a gene transcript set (GENCODE) but yielded no matches in the genome assembly were classified as exon–exon junction-spanning. For primer pairs with genomic matches, intron-spanning status was determined by comparing genomic and cDNA amplicon lengths.

## Data Availability

The original contributions presented in this study are included in the article/Supplementary Materials. Further inquiries can be directed to the corresponding author.

## Supplementary Materials

The following supporting information can be downloaded at: https://www.mdpi.com/article/10.3390/ijms27073156/s1. References [47,48] are cite in Supplementary Materials.
