# Supplementary material for: Intestinal Epithelial-like Cells Stimulated by a Functional Food Ingredient Promote Lysyl Oxidase (LOX) Expression in Osteoblast Precursor Cells via BMP-1 Secretion
Source: Int J Mol Sci. 2026 Mar 31;27(7):3156. doi: 10.3390/ijms27073156 (PMC13074062; doi:10.3390/ijms27073156)
Supplement: Supplementary file 1 [file ijms-27-03156-s001.zip › ijms-4146735-supplementary.pdf]

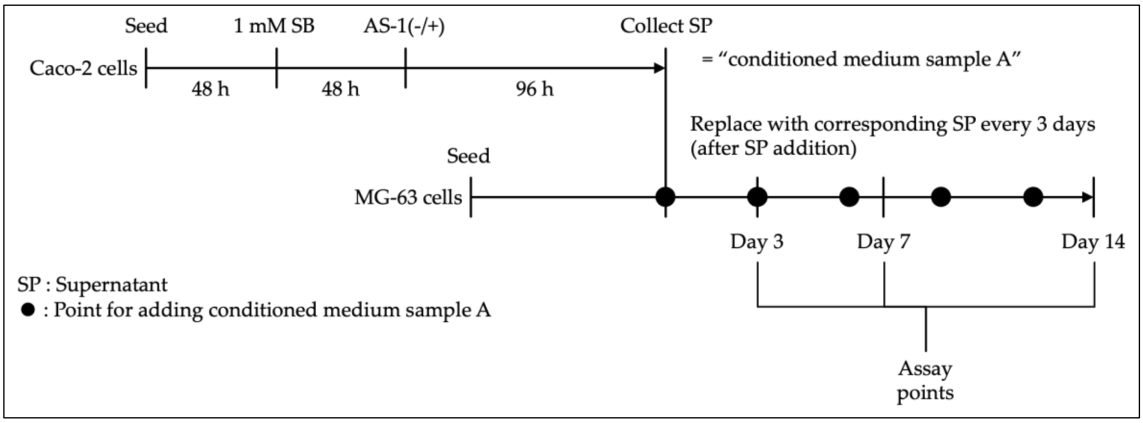

Supplementary Figure S1. Caco-2 cells were seeded and cultured for 48 h, then treated with 1 mM sodium butyrate (SB). After an additional 48 h, AS-1 was added. The Caco-2 supernatant collected after 96 h of culture following AS-1 addition was defined as SP(+) (total culture time: 192 h; total SB exposure time: 144 h). In parallel, an otherwise identical Caco-2 supernatant prepared without AS-1 was defined as SP(-). MG63 cells were seeded and, after 72 h, treated with SP(+) or SP(-); the medium was subsequently replaced every 3 days with the same corresponding SP(+) or SP(-). SP, supernatant. ●, time point for adding SP to MG63 cells.

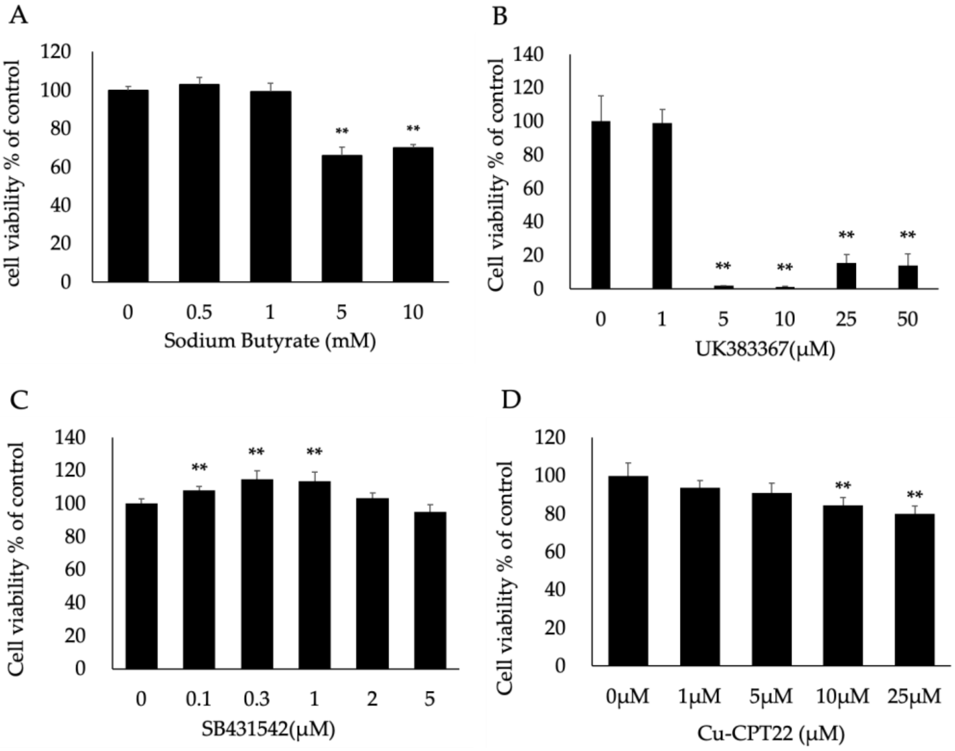

Supplementary Figure S2. Cell viability was assessed using the Cell Counting Kit-8 (CCK-8; WST-8–

based) assay to determine working concentrations of inhibitors. Cells were treated with the indicated compounds at the concentrations tested, and viable cells were quantified using the WST-8 assay. Based on these results, the concentrations used in subsequent experiments were set as follows: (A) Sodium Butyrate, 1 mM; (B) UK383367, 1 μM; (C) SB431542, 5 μM; (D) Cu-CPT22, 5 μM. These data are expressed as the mean ± SD (n=5 independent biological replicates). *: *p<0.05*, **: p<*0.01*; One-way ANOVA followed by Dunnett's multiple comparisons test (vs. control).

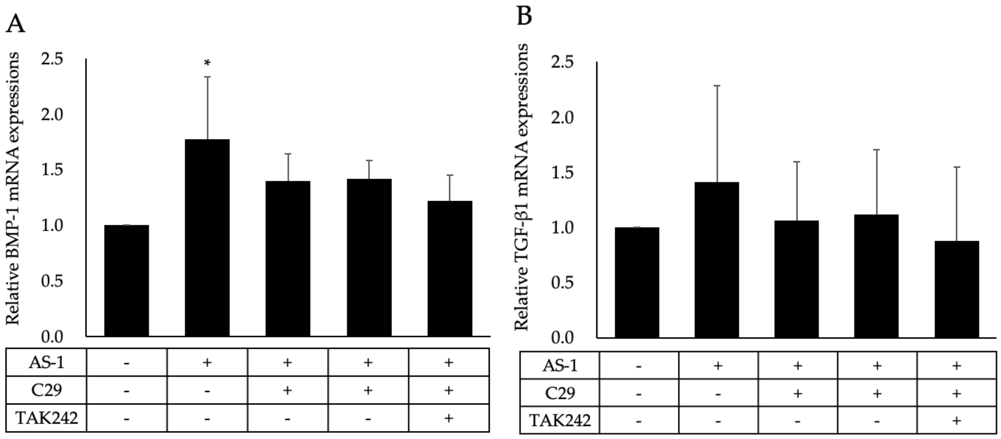

Supplementary Figure S3. Effects of TLR inhibitors (C29, a TLR2 inhibitor; TAK-242, a TLR4 inhibitor) on BMP-1 and TGF-β1 mRNA expression at 48 h after treatment. Cells were treated with Control, AS-1, AS-1 + C29, AS-1 + TAK-242, or AS-1 + C29 + TAK-242 (C29, 5 μM; TAK-242, 1 μM). The inhibitor concentrations were selected based on prior studies showing effective TLR antagonism in human cell systems, including Caco-2 monolayers and acceptable tolerability in Caco-2 cells (C29 [1], TAK-242[2]). mRNA expression was analyzed by qRT-PCR and normalized to RPL32. (A) BMP-1; (B) TGF-β1. Data are presented as mean ± SD (n=3 independent biological replicates). *: *p < 0.05* ; One-way ANOVA followed by Tukey's multiple comparisons test.

[1]. Mistry, P.; Laird, M.H.W.; Schwarz, R.S.; Greene, S.; Dyson, T.; Snyder, G.A.; Xiao, T.S.; Chauhan, J.; Fletcher, S.; Toshchakov, V.Y.; MacKerell, A.D., Jr.; Vogel, S.N. Inhibition of TLR2 Signaling by Small Molecule Inhibitors Targeting a Pocket within the TLR2 TIR Domain. Proc. Natl. Acad. Sci. U.S.A. 2015, 112, 5455–5460. https://doi.org/10.1073/pnas.1422576112

[2]. Ma, K.N.; Zhang, Y.; Zhang, Z.Y.; Wang, B.N.; Song, Y.Y.; Han, L.L.; Zhang, X.Z.; Long, S.R.; Cui, J.; Wang, Z.Q. Trichinella spiralis Galectin Binding to Toll-Like Receptor 4 Induces Intestinal Inflammation and Mediates Larval Invasion of Gut Mucosa. Vet. Res. 2023, 54, 113. https://doi.org/10.1186/s13567-023-01246-x

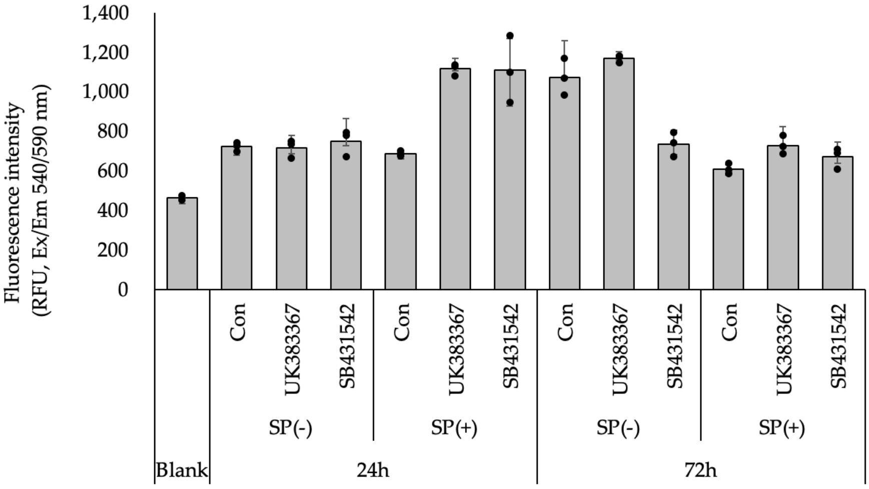

Supplementary Figure S4. Raw fluorescence intensity (RFU) before blank subtraction in the LOX activity assay (Ex/Em 540/590 nm; top read; gain optimized). Black dots indicate individual replicates (n=3). Blank was measured in duplicate (n=2). The same dataset was converted to LOX activity (µg/mL) using a standard curve after blank subtraction, as shown in Figure 4B.
